# Haemorrhagic event reporting with concomitant gemcitabine and warfarin: a VigiBase disproportionality analysis

**DOI:** 10.3389/fphar.2026.1858594

**Published:** 2026-09-10

**Authors:** Kazuki Nishida, Basile Chrétien, Angelique Da Silva, Aiko Calabia, Shigeyuki Matsui, Tomohiro Terada, Yuichi Ando

**Affiliations:** 1 Department of Biostatistics, Nagoya University Graduate School of Medicine, Nagoya, Aichi, Japan; 2 Department of Biostatistics and Data Science, School of Public Health, Kyoto University, Kyoto, Japan; 3 International Medical Education Department, Nagoya University Graduate School of Medicine, Aichi, Japan; 4 Normandie University, UNICAEN, INSERM U1086 ANTICIPE, Caen, France; 5 PICARO Cardio-Oncology Program, Departments of Pharmacology and Medical Oncology, Caen-Normandy University Hospital, Caen, France; 6 Department of Clinical Pharmacology and Therapeutics, Kyoto University Hospital, Kyoto, Japan; 7 Department of Clinical Oncology and Chemotherapy, Nagoya University Hospital, Nagoya, Japan

**Keywords:** drug-drug interaction, gemcitabine, haemorrhage, pharmacovigilance, warfarin

## Abstract

**Purpose:**

Case reports suggest that concomitant gemcitabine and warfarin may lead to clinically important over-anticoagulation and bleeding, but population-level characterization remains limited. We evaluated whether concomitant gemcitabine and oral anticoagulants, particularly warfarin, were associated with disproportionate reporting of haemorrhagic events in VigiBase, the WHO global database of individual case safety reports.

**Methods:**

We performed a retrospective pharmacovigilance study of VigiBase reports from 1 January 1994 to 28 April 2025. Haemorrhagic events were defined using the Standardised MedDRA Query “Haemorrhages”. For each anticoagulant, we fitted multivariable logistic regression models including a gemcitabine × anticoagulant interaction term, adjusting for age group, sex, WHO region, and bleeding-related co-medications. A sensitivity analysis was restricted to serious reports. Additional analyses adjusted for calendar period. Time-to-onset and dechallenge/rechallenge were summarised where available.

**Results:**

Among 6,191,806 eligible reports, 707 described concomitant gemcitabine and warfarin. The gemcitabine–warfarin interaction term was significantly positive (interaction reporting odds ratio 1.31, 95% confidence interval 1.07–1.60; p = 0.010) and remained positive in serious reports (1.36, 1.09–1.69; p = 0.006). Interaction-term estimates for direct oral anticoagulants were inconsistent or not robust in sensitivity analyses. Time-to-onset was available for 29 haemorrhagic reports, with a median of 15 days (interquartile range 10–52; range 4–529 days). Positive dechallenge was recorded in 17 reports; no positive rechallenge was recorded.

**Conclusion:**

This VigiBase analysis identified disproportionate haemorrhagic-event reporting with concomitant gemcitabine and warfarin. These findings should be interpreted in light of the limitations inherent to spontaneous reporting, including under-reporting, reporting biases, missingness, and residual confounding.

## Introduction

Warfarin is widely used for the prevention and treatment of thromboembolic disease and requires careful dose adjustment because its anticoagulant response varies substantially across patients and is highly sensitive to changes in exposure and vitamin K balance. As a result, clinically meaningful drug–drug interactions frequently manifest as supratherapeutic international normalized ratio (INR) values and bleeding risk, and INR monitoring is central to safe therapy (Holford, 1986).

Patients with cancer are particularly vulnerable to unstable anticoagulation control. Malignancy is associated with a hypercoagulable state and a high burden of thrombotic complications, while anticancer therapy and supportive care introduce frequent changes in concomitant medications, organ function, and nutritional intake—each of which can perturb warfarin dose–response. In this context, identifying chemotherapy-associated disturbances in warfarin control is clinically important because unexpected INR elevation can cause major bleeding and may interrupt oncologic treatment (Zwicker et al., 2007).

Among antimetabolite chemotherapy agents, fluoropyrimidines are a well-established example of clinically significant potentiation of warfarin. A consecutive case series described marked INR elevations and substantial warfarin dose reductions in patients receiving 5-fluorouracil with warfarin, including serious bleeding events (Kolesar et al., 1999). Subsequent clinical studies and retrospective cohorts further demonstrated substantial INR increases and bleeding risk with concomitant capecitabine or 5-fluorouracil in warfarin-treated patients, supporting the need for intensified INR monitoring during fluoropyrimidine-based chemotherapy (Shah et al., 2010; Shah et al., 2006).

In contrast, the interaction between gemcitabine and warfarin has remained less clearly characterized despite repeated clinical observations. The earliest detailed report described a patient stable on warfarin whose INR rose promptly after gemcitabine initiation, decreased during the rest period, and increased again on gemcitabine re-exposure, necessitating warfarin dose reductions and returning to baseline after gemcitabine discontinuation—features suggestive of a temporal and potentially causal relationship (Kinikar and Kolesar, 1999). Shortly thereafter, a manufacturer correspondence discussed a limited number of suspected cases in post-marketing safety data, leading to uncertainty about the overall frequency and strength of association (Kilgour-Christie and Czarnecki, 2002).

Since these initial publications, additional case reports have continued to describe supratherapeutic INR values and bleeding in warfarin-treated patients receiving gemcitabine-based therapy, including gastrointestinal bleeding with markedly elevated INR, as well as difficulty maintaining stable anticoagulation during gemcitabine exposure (Saif, 2005; Yoshioka et al., 2012). Importantly, a clinically significant INR increase has also been reported following intravesical gemcitabine administration, suggesting that the phenomenon may occur even outside standard systemic dosing contexts (Kurtzhalts et al., 2016). At the same time, contemporary cases highlight the complexity of attributing causality in oncology settings where multiple interacting factors—including co-administered anti-cancer agents and complementary medicines—may contribute to warfarin over-anticoagulation (Moussouni et al., 2023). Collectively, the existing literature supports the plausibility of a gemcitabine–warfarin interaction but remains dominated by case-based evidence and lacks a robust, population-level quantitative assessment.

Global pharmacovigilance databases provide an opportunity to evaluate such safety signals at scale. VigiBase, the World Health Organization global database of individual case safety reports, is widely used for pharmacovigilance signal detection and characterization (Brand et al., 2026). Disproportionality approaches can assess whether specific drug–event combinations are reported more frequently than expected within spontaneous reporting systems (Evans et al., 2001; Bate et al., 1998; Norén et al., 2013). Using this framework, it is possible to evaluate haemorrhagic-event reporting associated with concomitant gemcitabine and oral anticoagulants and to describe clinical and reporting features of the relevant reports.

Accordingly, the present study aimed to evaluate haemorrhagic-event reporting associated with concomitant gemcitabine and oral anticoagulants, particularly warfarin, in VigiBase, and to characterize report attributes relevant to clinical interpretation, including seriousness, clinical outcomes, and temporal patterns where available.

## Methods

### Data source and study design

We conducted a retrospective pharmacovigilance study using VigiBase, the World Health Organization (WHO) global database of individual case safety reports (ICSRs), managed by the Uppsala Monitoring Centre (UMC). The design and reporting of this disproportionality analysis were guided by the READUS-PV recommendations for studies using individual case safety reports (Fusaroli et al., 2024a; Fusaroli et al., 2024b). The analysis was performed on the VigiBase Extract Case Level dataset provided by UMC (each report identified by a unique UMCReportID). Medicinal products are coded using WHODrug Global and adverse reactions are coded using MedDRA. The unit of analysis for the main models was the ICSR (case level). Drug–reaction pair–level fields were used for time-to-onset and dechallenge/rechallenge descriptions.

We selected VigiBase because it is the largest global repository of individual case safety reports, aggregating tens of millions of reports contributed by more than 130 countries participating in the WHO Programme for International Drug Monitoring, with standardised WHODrug Global and MedDRA coding that enables consistent identification of drug and reaction terms across 3 decades and across diverse healthcare settings (Brand et al., 2026). This global scale was important for the present question, because concomitant exposure to gemcitabine and an oral anticoagulant is uncommon and would yield too few informative reports in any single national system; the breadth of VigiBase also supported the anticoagulant-by-anticoagulant comparison that is central to our analysis. We considered alternative data sources, including the United States FDA Adverse Event Reporting System (FAERS), EudraVigilance, and administrative claims or electronic health-record databases. National spontaneous-reporting systems such as FAERS are largely encompassed by VigiBase and would have restricted geographical coverage, whereas claims and health-record databases offer exposure denominators and richer confounder adjustment but capture too few gemcitabine–anticoagulant co-exposures to support the present comparison and lack the global scope required for rare drug–drug-interaction signal detection. The principal disadvantages of VigiBase are those intrinsic to spontaneous reporting—under-reporting, differential and notoriety-related reporting, absence of an exposure denominator, potential duplicate reports, and frequent missingness of clinical detail (including INR values and laboratory data)—which we consider further in the Discussion.

### Eligibility and analytic datasets

We included reports between 1st January 1994 (first year with gemcitabine reports) until 28th April 2025 with available information on patient sex, age group, and reporting region (WHO regions), to allow covariate adjustment. The primary multivariable models were fitted on 6,191,806 reports. A sensitivity analysis restricted to serious haemorrhage reports at the case level, regardless of the reported reaction, was fitted on 6,171,120 reports. In this analysis, haemorrhage reports were then evaluated using the same SMQ-based outcome definition as in the primary analysis. In pharmacovigilance, a report is considered serious when the adverse event associated with a medical product leads to death, is life-threatening, results in hospitalization or prolonged hospitalization, causes disability or permanent damage, results in a congenital anomaly/birth defect, or requires medical or surgical intervention to prevent permanent impairment or other important medical outcomes (U.S. Food and Drug Administration, 2024).

### Exposure definition

The exposure of interest was gemcitabine, defined as the presence of gemcitabine in the report drug list. Oral anticoagulants evaluated as co-exposures were warfarin, acenocoumarol, phenprocoumon, fluindione, rivaroxaban, apixaban, edoxaban, and dabigatran, each defined as presence in the report drug list. Drugs were considered irrespective of role (suspected, interacting, or concomitant) as recorded in VigiBase. In a sensitivity analysis, gemcitabine exposure was restricted to reports in which it was recorded as a suspected or interacting drug (i.e., excluding reports in which gemcitabine was coded only as concomitant), to assess whether the interaction signal was robust to indication bias arising from incidental co-listing. Parenteral anticoagulants, namely, low-molecular-weight heparins (LMWH) and unfractionated heparin (UFH), were purposely excluded from this analysis. This decision was based on the absence of a plausible pharmacokinetic or pharmacodynamic drug-drug interaction (DDI) pathway with gemcitabine that would exacerbate bleeding risk, in contrast to the theoretical and documented interaction mechanisms concerning oral anticoagulants.

### Outcome definition

The outcome was reporting of haemorrhagic events, defined at the case level as the presence of at least one MedDRA reaction term belonging to the broad Standardised MedDRA Query (SMQ) “Haemorrhages” (See Supplementary Table 1 for details). For descriptive analyses at the drug–reaction pair level, the same SMQ definition was applied to identify gemcitabine–haemorrhage pairs.

### Covariates

All multivariable models adjusted for patient sex, age group (VigiBase age group categories), reporting WHO region, and several co-medications. To mitigate confounding by co-medications plausibly associated with inherent bleeding risk, altered hemostasis, and/or anticoagulation instability, we included binary indicators for selected co-reported medication classes. These comprised agents directly impairing hemostasis or mucosal integrity, including platelet inhibitors (B01AC ATC codes) and non-steroidal anti-inflammatory drugs (NSAIDs, M01A ATC codes) because of their antiplatelet effects and gastrointestinal toxicity (Parekh et al., 2015; Gladding et al., 2008), systemic corticosteroids (H02 ATC codes) because of their association with capillary fragility (Ullian, 1999) and gastrointestinal bleeding (Narum et al., 2014), and SSRIs/SNRIs (N06AA and N06AB ATC codes), which may impair platelet aggregation via serotonin depletion (Grech et al., 2022). We also adjusted for antineoplastic agents frequently co-prescribed with gemcitabine that may increase bleeding risk through myelosuppression, particularly thrombocytopenia, or mucosal toxicity. These included taxanes (L01CD ATC codes) (Salem et al., 2023) and platinum-based anticancer drugs (L01XA ATC codes) (Oun et al., 2018). In addition, fluoropyrimidines (capecitabine/5-fluorouracil; L01BC06, L01BC02, and L01BC52 ATC codes) were included because of their established pharmacokinetic interactions with vitamin K antagonists, which may precipitate marked anticoagulation instability (Giunta, 2010).

### Statistical analysis

We assessed whether concomitant gemcitabine and each oral anticoagulant were associated with excess haemorrhagic-event reporting using multivariable logistic regression models. For each anticoagulant, a separate model was fitted with haemorrhagic-event reporting (yes/no) as the dependent variable and gemcitabine exposure, anticoagulant exposure, and their product term (gemcitabine × anticoagulant) as key independent variables, together with the covariates described above. Exponentiated coefficients were expressed as adjusted reporting odds ratios (aRORs) with Wald 95% confidence intervals. The exponentiated coefficient of the product term was interpreted as an interaction reporting odds ratio (IROR), representing the multiplicative departure from the reporting odds expected under independent effects of gemcitabine and the anticoagulant. Statistical significance was evaluated using two-sided tests with α = 0.05. Warfarin was pre-specified as the primary comparison, on the basis of prior case-report evidence of a gemcitabine–warfarin interaction; analyses of the other anticoagulants, including the direct oral anticoagulants, were considered exploratory. To address multiple testing across the eight anticoagulants, we additionally report Bonferroni-corrected p-values and Benjamini–Hochberg false-discovery-rate q-values for the interaction terms (Supplementary Table 3). Because the study period spanned 3 decades, over which reporting practices and the anticoagulant landscape evolved, we performed an additional sensitivity analysis in which each model was further adjusted for calendar period, categorised by the year in which the report first entered VigiBase (1994–2005, 2006–2011, 2012–2017, and 2018–2025; Supplementary Table 4). All analyses were performed in R version 4.6.0 (R Foundation for Statistical Computing, Vienna, Austria), using the vigicaen and data.table packages. The analysis code is available from the corresponding author on reasonable request. The study protocol was not registered.

To evaluate robustness in clinically more consequential reports, the same modelling strategy was repeated after restricting the analytic dataset to serious reports (Serious = “Y”). Time-to-onset (TTO) was derived from VigiBase LINK-table fields (TimeToOnsetMin/Max), representing the interval between drug start date and reaction start date in days, and was summarised using TimeToOnsetMax as the median, interquartile range, and overall range among drug–reaction pairs with available data. Positive dechallenge was defined as recorded improvement or abatement of the reaction after withdrawal of gemcitabine in the gemcitabine–haemorrhage pair. Rechallenge information was summarised descriptively, as was gemcitabine indication. All descriptive analyses used an available-case approach because these fields are frequently missing in spontaneous reports.

## Results

### Study population and report characteristics

The study included 6,191,806 individual case safety reports (ICSRs) in VigiBase from January 1994 to April 2025. Of these, 107,530 reports included gemcitabine and 62,574 included warfarin. Concomitant exposure to gemcitabine and warfarin was reported in 707 ICSRs.

Characteristics of ICSRs with concomitant gemcitabine–warfarin exposure (n = 707) are summarised in Table 1. Reports predominantly concerned older individuals, with 51% aged 65 years or older (median age category 45–64). Sex distribution was nearly balanced (52% male). Most reports originated from the Americas (n = 567, 80%), followed by the Western Pacific (n = 93, 13%). Within these reports, haemorrhagic events were reported in 180 cases (25.5%). The majority were classified as serious (90.8% among those with known seriousness).

**TABLE 1:** Characteristics of individual case safety reports in VigiBase.

Characteristic	Gemcitabine (n = 107,530)	Warfarin (n = 62,574)	Gemcitabine + warfarin (n = 707)
Age group, n (%)			
0–17 years	548 (0.5%)	203 (0.5%)	0 (0%)
18–45 years	8,832 (9.7%)	3,340 (7.5%)	30 (5.6%)
45–64 years	43,804 (48.0%)	13,602 (31.0%)	234 (43.0%)
65–74 years	27,627 (30.0%)	13,160 (30.0%)	164 (30.0%)
75+ years	10,483 (11.0%)	14,250 (32.0%)	111 (21.0%)
Unknown	16,236	18,019	168
Sex, n (%)			
Male	51,441 (51.0%)	28,615 (48.0%)	353 (52.0%)
Female	49,633 (49.0%)	31,406 (52.0%)	326 (48.0%)
Unknown	6,456	2,553	28
WHO region, n (%)			
Americas	24,915 (23.0%)	49,449 (79.0%)	567 (80.0%)
European	25,165 (23.0%)	7,889 (13.0%)	40 (5.7%)
Western Pacific	47,129 (44.0%)	4,844 (7.7%)	93 (13.0%)
Other regions	10,321 (9.5%)	392 (0.6%)	7 (1.0%)
Event reporting, n (%)			
Haemorrhagic event	4,886 (4.5%)	13,076 (21.0%)	180 (25.5%)
Serious reports^a^	71,060 (71.0%)	42,027 (73.0%)	540 (90.8%)
Clinical outcomes^b^, n (%)			
Death	—	—	144 (31.0%)
Hospitalization	—	—	213 (45.0%)

^a^ Percentage calculated among reports with known seriousness.

^b^ Outcomes provided for the concomitant exposure group where specific outcome fields were populated.

Across all gemcitabine-involving reports, the most frequently co-reported medications were other cytotoxic agents, including cisplatin (22.2%), carboplatin (14.4%), and paclitaxel (13.3%) (Table 2). In reports with concomitant gemcitabine–warfarin exposure, platinum-based agents and taxanes were co-reported in 32% and 27% of reports, respectively.

**TABLE 2:** Top most frequently co-reported medications in gemcitabine ICSRs.

Rank	Medication	Count (N)	Frequency (%)
1	Cisplatin	23,865	22.20%
2	Carboplatin	15,431	14.40%
3	Paclitaxel	14,354	13.30%
4	Dexamethasone	9,203	8.60%
5	Sodium chloride	6,876	6.40%
6	Oxaliplatin	6,494	6.00%
7	Docetaxel	5,202	4.80%
8	Ondansetron	4,771	4.40%
9	Fluorouracil	4,330	4.00%

ICSR, individual case safety report.

Among the total reports involving gemcitabine, the specific indication for use was frequently unreported or classified as unknown (N = 53,364), a common limitation of spontaneous reporting systems. When clinical indications were available and mapped to the MedDRA (Medical Dictionary for Regulatory Activities) High-Level Term (HLT) hierarchy, the most frequently reported underlying condition was pancreatic neoplasms (N = 14,137). Other prominent known indications included malignant hepatobiliary neoplasms (N = 5,782), malignant breast and nipple neoplasms (N = 4,422), and specified non-small cell malignant neoplasms of the respiratory tract (N = 3,844) (Table 3).

**TABLE 3:** Top 20 reported indications for gemcitabine.

Indication (MedDRA HLT)	All gemcitabine cases (N, %)	Co-prescription with warfarin (N, %)
Unknown	53,364 (49.6%)	295 (41.7%)
Pancreatic neoplasms	14,137 (13.1%)	123 (17.4%)
Malignant hepatobiliary neoplasms	5,782 (5.4%)	25 (3.5%)
Breast and nipple neoplasms malignant	4,422 (4.1%)	30 (4.2%)
Non-small cell neoplasms malignant of the respiratory tract cell type specified	3,844 (3.6%)	55 (7.8%)
Respiratory tract and pleural neoplasms malignant cell type unspecified NEC	3,112 (2.9%)	13 (1.8%)
Ovarian neoplasms malignant (excl germ cell)	3,069 (2.9%)	16 (2.3%)
Bladder neoplasms malignant	3,023 (2.8%)	29 (4.1%)
Therapeutic procedures NEC	1,892 (1.8%)	35 (5.0%)
Neoplasms malignant site unspecified NEC	1,733 (1.6%)	12 (1.7%)
Malignant musculoskeletal and connective tissue neoplasms	1,184 (1.1%)	9 (1.3%)
Urinary tract neoplasms malignant NEC	1,162 (1.1%)	10 (1.4%)
Diffuse large B-cell lymphomas	1,135 (1.1%)	3 (0.4%)
Chemotherapies	1,110 (1.0%)	7 (1.0%)
Hodgkin’s disease NEC	620 (0.6%)	1 (0.1%)
Neoplasms unspecified malignancy and site unspecified NEC	408 (0.4%)	1 (0.1%)
Renal pelvis and ureter neoplasms malignant	405 (0.4%)	3 (0.4%)
Malignant intestinal neoplasms	399 (0.4%)	6 (0.8%)
Non-Hodgkin’s lymphomas NEC	363 (0.3%)	1 (0.1%)
Renal neoplasms malignant	355 (0.3%)	9 (1.3%)

Abbreviations: MedDRA, medical dictionary for regulatory activities; HLT, High-Level Term; NEC, not elsewhere classified; N, number of cases.

Data Source: Data were extracted from VigiBase, the World Health Organization (WHO) global database of individual case safety reports. Reported indications for drug use were mapped to the MedDRA, High-Level Term (HLT) hierarchy to group similar clinical conditions.

Missing Data (“Unknown”): The “Unknown” category represents cases where the indication for gemcitabine was not provided by the original reporter. Missing or incomplete data is a well-known characteristic of spontaneous pharmacovigilance reporting systems.

Methodology: If a single case report contained multiple specific indications that mapped to the exact same MedDRA HLT (e.g., “Pancreatic cancer” and “Pancreatic carcinoma”), the case was only counted once within that HLT, to avoid artificial duplication of patient counts.

This distribution of clinical indications remained largely consistent among the sub-groups of patients co-prescribed with anticoagulants. Within the cohort receiving concomitant warfarin, pancreatic neoplasms (N = 123) and non-small cell lung neoplasms (N = 55) represented the most common known indications. Similar trends were observed across the direct oral anticoagulants (DOACs); for example, pancreatic neoplasms remained the leading documented indication for patients co-prescribed apixaban (N = 93) and rivaroxaban (N = 78) (Supplementary Table 2).

### Interaction signal quantification

Multivariable logistic regression models adjusted for age, sex, region, and co-reported medication classes associated with bleeding included a gemcitabine × warfarin interaction term that was statistically significant on the reporting odds scale (Table 4). The adjusted Interaction Reporting Odds Ratio (IROR) for gemcitabine–warfarin was 1.31 (95% CI 1.07–1.60; p = 0.010), indicating excess haemorrhagic-event reporting beyond that expected under a multiplicative model based on the individual reporting-odds associations of each drug. Findings were similar in the sensitivity analysis restricted to serious reports (IROR 1.36, 95% CI 1.09–1.69; p = 0.006).

**TABLE 4:** Interaction signal for haemorrhagic events (adjusted logistic regression).

Anticoagulant	Primary analysis (adjusted IROR, 95% CI)	Serious-report analysis (adjusted IROR, 95% CI)
Acenocoumarol	0.41 (0.15–1.18)	0.25 (0.06–1.04)
Apixaban	1.40 (0.98–2.00)	1.23 (0.85–1.78)
Dabigatran	0.39 (0.15–1.03)	0.36 (0.14–0.95)
Edoxaban	1.74 (1.05–2.88)	1.32 (0.77–2.26)
Fluindione	0.70 (0.34–1.46)	0.64 (0.29–1.41)
Phenprocoumon	1.34 (0.68–2.65)	0.90 (0.40–2.03)
Rivaroxaban	0.86 (0.60–1.24)	0.71 (0.49–1.04)
Warfarin	1.31 (1.07–1.60)	1.36 (1.09–1.69)

IROR, interaction reporting odds ratio.

Models were adjusted for age, sex, WHO region, and co-administration of antiplatelets, NSAIDs, corticosteroids, SSRI/SNRIs, fluoropyrimidines, taxanes, and platinum-based agents.

In a sensitivity analysis restricting gemcitabine to a suspected or interacting role (excluding 13,638 reports, 12.7%, in which gemcitabine was coded only as concomitant; 495 of the 707 gemcitabine–warfarin co-exposed reports were retained), the gemcitabine–warfarin interaction was essentially unchanged (IROR 1.28, 95% CI 1.00–1.63 for all haemorrhagic events; IROR 1.33, 95% CI 1.02–1.72 for serious reports), indicating that the signal was not driven by incidental concomitant co-listing (Supplementary Table 3). After correction for multiple testing across the eight anticoagulants, the warfarin interaction remained significant in the serious-report analysis (Bonferroni-corrected p = 0.046; Benjamini–Hochberg q = 0.046) and was attenuated to a non-significant trend in the primary all-report analysis (Bonferroni-corrected p = 0.081), although the direction and magnitude of the estimate were stable across all analyses. No other anticoagulant showed a consistent interaction signal after correction for multiple testing.

Reporting of haemorrhagic events within the study population declined over calendar time, from 7.7% of oncology reports in 1994–2005 to 4.3% in 2018–2025. After adjustment for calendar period, the gemcitabine–warfarin interaction was essentially unchanged for all haemorrhagic events (IROR 1.24, 95% CI 1.01–1.52) and, for serious reports, remained statistically significant, including after correction for multiple testing (IROR 1.42, 95% CI 1.14–1.77; Bonferroni-corrected p = 0.013; Benjamini–Hochberg q = 0.013; Supplementary Table 4). The signal remained specific to warfarin, with no consistent interaction for the direct oral anticoagulants, arguing against the association being an artefact of secular changes in reporting practice.

**FIGURE 1:**
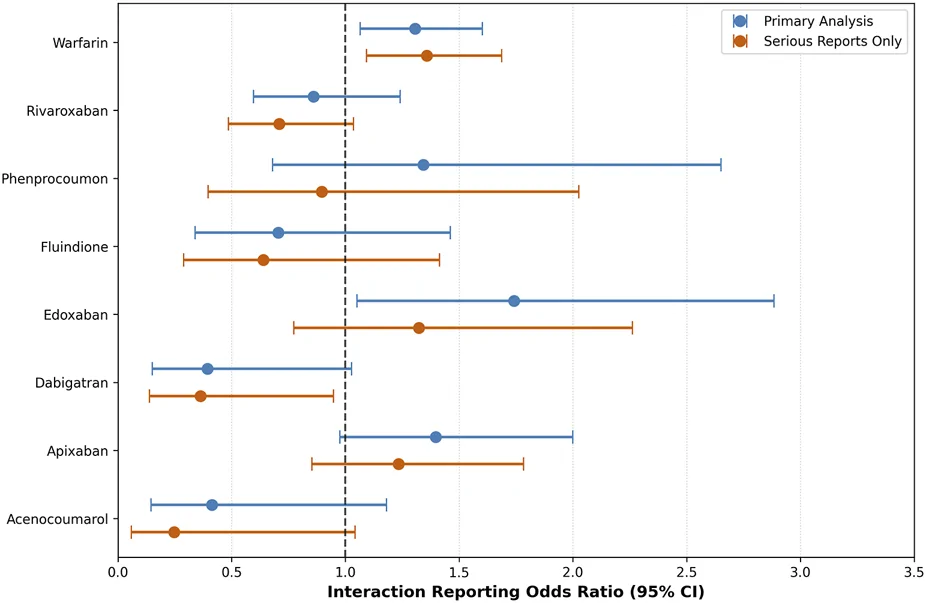
Interaction reporting odds ratios (IRORs) for haemorrhagic events associated with concomitant gemcitabine and oral anticoagulants in VigiBase. Forest plot showing adjusted IRORs with 95% confidence intervals derived from multivariable logistic regression models including a product term (gemcitabine × anticoagulant). Models were adjusted for age group, sex, WHO region, and co-reported medication classes associated with bleeding risk (antiplatelets, NSAIDs, systemic corticosteroids, SSRI/SNRIs, fluoropyrimidines, taxanes, and platinum-based agents). Blue circles represent the primary analysis including all reports; orange circles represent the sensitivity analysis restricted to serious reports. The vertical dashed line indicates the null value (IROR = 1). A significant positive interaction signal was observed for warfarin in both analyses, whereas other anticoagulants did not demonstrate a consistent interaction signal.

In contrast, other oral anticoagulants did not show a consistent statistical interaction-term signal on the reporting odds scale (Figure 1). In the primary analysis, the edoxaban interaction-term estimate was above 1.0 (IROR 1.74, 95% CI 1.05–2.88), but this was not replicated in the serious-report sensitivity analysis (IROR 1.32, 95% CI 0.77–2.26). For rivaroxaban and apixaban, as well as for other vitamin K antagonists such as acenocoumarol, phenprocoumon, and fluindione, interaction-term estimates were not statistically significant. The dabigatran interaction-term estimate was nominally inverse in the serious-report analysis (IROR 0.36, 95% CI 0.14–0.95), but this did not persist after correction for multiple testing (Bonferroni-corrected p = 0.31; Benjamini–Hochberg q = 0.15) and was not seen in the primary analysis (IROR 0.39, 95% CI 0.15–1.03).

### Temporal analysis and case outcomes

**FIGURE 2:**
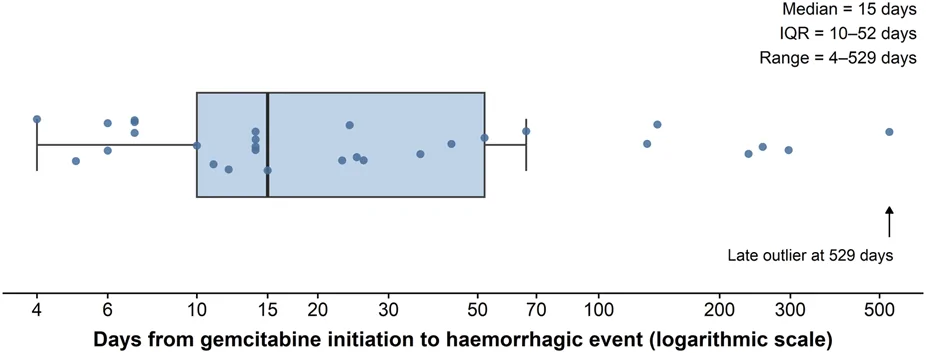
Time-to-onset of haemorrhagic events following initiation of gemcitabine among haemorrhagic reports with concomitant gemcitabine and warfarin exposure (n = 29 with available data). Box-and-whisker plot with overlaid individual data points showing the distribution of time-to-onset (days) calculated using TimeToOnsetMax from the VigiBase LINK table. The horizontal axis is on a logarithmic scale; all boxplot statistics were calculated on the original day scale. The box represents the interquartile range (IQR), the central line indicates the median, and whiskers represent the range excluding outliers. The median time-to-onset was 15 days (IQR 10–52 days; range 4–529 days). The most extreme observation, at 529 days, is annotated.

Of the 180 haemorrhagic ICSRs with concomitant exposure, time-to-onset (TTO) data were available for 29. Based on the recorded gemcitabine start date and reaction onset date fields, the median TTO was 15 days (IQR 10–52), with reported intervals ranging from 4 to 529 days (Figure 2). Because TTO was missing for 84% of the 180 haemorrhagic reports (available for 29; 16%), these values are descriptive and based on available reports only, and should not be interpreted as a definitive early-risk window. The single late value of 529 days probably reflects prolonged or maintenance gemcitabine exposure, delayed reporting, or imprecise date recording rather than an acute event.

Outcome information was available for a subset of reports. Among all reports involving the co-administration of gemcitabine and warfarin—irrespective of whether the reported adverse event was a haemorrhage or another reaction—death was recorded in 31% and hospitalization in 45% of those with documented outcomes. Dechallenge information for haemorrhage indicated that 17 reports were classified as positive dechallenge, with improvement after gemcitabine discontinuation recorded for these reports. Rechallenge data were documented in 11 reports; none were classified as positive rechallenge.

## Discussion

Using multivariable models incorporating interaction terms, we observed a statistically significant and reproducible gemcitabine × warfarin interaction term on the reporting odds scale. Reports involving this combination showed excess haemorrhagic-event reporting beyond that expected under a multiplicative model based on the individual reporting-odds associations of each drug. The gemcitabine × warfarin interaction-term estimate on the reporting odds scale was statistically significant in the primary analysis (IROR 1.31, 95% CI 1.07–1.60) and was similar in the sensitivity analysis restricted to serious reports (IROR 1.36, 95% CI 1.09–1.69). Whereas previous clinical observations have often considered anticoagulants as a group (Kilgour-Christie and Czarnecki, 2002), the present analysis indicates that the interaction-term signal within this reporting system was more consistently observed for warfarin than for other oral anticoagulants. The absence of a comparable signal with DOACs should be interpreted cautiously, as differences in prescribing patterns, underlying indications, and reporting behaviour may influence disproportionality estimates. Clinically, this distinction is relevant because patients receiving gemcitabine often have fluctuating nutritional status, hepatic reserve, concomitant medications, and treatment-related complications that can make stable warfarin management particularly difficult. In this setting, disproportionate haemorrhagic-event reporting with warfarin may be meaningful for treatment monitoring and supportive care in routine oncology practice.

The excess haemorrhagic-event reporting observed with concomitant gemcitabine and warfarin exposure likely reflects multiple, interacting mechanisms operating within a clinically complex oncology setting. Although gemcitabine is not generally considered a potent direct inhibitor of cytochrome P450 (CYP) enzymes (de Sousa Cavalcante and Monteiro, 2014), the observed interaction-term signal may reflect multiple contributory pathways, including pharmacokinetic, pharmacodynamic, and patient-context factors. In oncology patients, systemic inflammation, treatment-related hepatic dysfunction, and hypoalbuminaemia may reduce metabolic capacity or increase the free fraction of warfarin, destabilising anticoagulation (Rivory et al., 2002; Behranvand et al., 2022; Gemcitabine, 2012; Strople et al., 2009; Kawai et al., 2019). Several features of warfarin may render it uniquely susceptible to such perturbations relative to the direct oral anticoagulants. Warfarin has a narrow therapeutic index and is titrated to a laboratory target (the INR), so that even modest changes in clearance or in vitamin K balance can translate into measurable over-anticoagulation (Johnson, 2012); it is administered as a racemate whose more potent S-enantiomer is metabolised by CYP2C9 (Flora et al., 2017); it is extensively (approximately 99%) bound to plasma albumin; and its effect depends on the hepatic synthesis of vitamin K–dependent clotting factors (Ganeval et al., 1986). Gemcitabine-associated systemic inflammation and hepatic dysfunction (Gemcitabine, 2012), which downregulate CYP activity, hypoalbuminaemia, which raises the free warfarin fraction, and reduced vitamin K intake or absorption during chemotherapy would therefore be expected to destabilise warfarin control preferentially. By contrast, the direct oral anticoagulants have wider therapeutic windows, are given at fixed doses without routine coagulation monitoring, depend less on CYP2C9 and on vitamin K status, and are less extensively protein-bound—properties consistent with the compound-specific pattern observed here. Bleeding susceptibility may also be influenced by gemcitabine-related myelosuppression (including thrombocytopenia) and rare endothelial/vascular toxicities, which could increase haemorrhage risk independent of INR (Cassidy et al., 2001; Dasanu, 2008). The distribution of reported time-to-onset (TTO) values showed a median of 15 days (IQR 10–52), with some reported intervals falling within the first week after the recorded start date of gemcitabine therapy. Dechallenge information was classified as positive in 17 reports, indicating that improvement after gemcitabine discontinuation was recorded in those cases. Although temporal proximity and dechallenge patterns may be consistent with a temporal association in a subset of reports, these observations do not establish causality. In particular, positive dechallenge supports a temporal association but does not, on its own, confirm a specific drug–drug interaction mechanism, because improvement after gemcitabine withdrawal may also reflect warfarin dose reduction, vitamin K administration, or blood or platelet transfusion. Nonetheless, the early and relatively consistent time-to-onset in the subset with available data, together with positive dechallenge in 17 reports, are the reporting features one would expect of a genuine pharmacological effect and are difficult to reconcile with a purely spurious association. Notably, the earliest detailed report of this interaction documented a positive rechallenge, with the INR rising again upon gemcitabine re-exposure and returning towards baseline after discontinuation (Kinikar and Kolesar, 1999), providing individual-level temporal evidence that complements the aggregate dechallenge pattern observed in the present dataset.

Interpretation should also account for the limitations of spontaneous reporting data. Although we adjusted for several co-reported medication classes, residual confounding from co-medications and underlying clinical factors cannot be excluded. Under-reporting, differential reporting, and possible notoriety bias may have influenced reporting of the gemcitabine–warfarin combination. Reporting was also geographically concentrated, with most reports originating from the Americas, where warfarin is the predominant oral vitamin K antagonist, whereas the other coumarins we evaluated (acenocoumarol, phenprocoumon, and fluindione) are prescribed largely in particular European regions and contributed comparatively few co-exposed reports. The apparent specificity of the signal to warfarin should therefore be interpreted with caution, because the null estimates for the other vitamin K antagonists may partly reflect limited statistical power and regional prescribing patterns rather than a true absence of interaction; a class effect across vitamin K antagonists cannot be excluded, and dedicated evaluation in regions where these agents are commonly used would help to distinguish a warfarin-specific from a coumarin-class interaction. The absence of an exposure denominator precludes estimation of absolute risk, and the IROR should therefore be interpreted as a measure of disproportionate reporting rather than clinical risk. Report-level missingness and limited clinical detail, including INR values, cancer stage, liver function, and nutritional status, also constrain mechanistic interpretation. Accordingly, these findings indicate a reproducible statistical signal but do not establish a direct causal drug–drug interaction. Three features nonetheless argue against the signal being a mere artefact of indication or disease-severity confounding. First, restricting gemcitabine to a suspected or interacting role left the warfarin interaction essentially unchanged (Supplementary Table 3), making indication bias from incidental concomitant co-listing an unlikely sole explanation. Second, the signal was specific to warfarin, whereas the direct oral anticoagulants were null; if generic cancer severity or combination chemotherapy were driving the result, a comparable interaction would be expected across anticoagulants, and the distribution of high-risk indications (e.g., pancreatic and hepatobiliary neoplasms) was in fact similar across the warfarin and DOAC sub-groups. Third, the gemcitabine–warfarin interaction was essentially unchanged after adjustment for calendar period, indicating that secular changes in reporting practice over the three-decade study period are unlikely to account for the signal.

Taken together, the persistence of the signal in the serious-report analysis, the early reported time-to-onset in the subset with available data, and positive dechallenge in a subset of reports support heightened clinical vigilance when gemcitabine and warfarin are co-administered, including the period shortly after gemcitabine initiation, while recognising the limited availability of time-to-onset data. However, these findings should be interpreted as hypothesis-generating and do not establish causality or absolute risk.

## Conclusion

Our VigiBase analysis identified disproportionate haemorrhagic-event reporting with concomitant gemcitabine and warfarin. Although these findings do not establish causality or absolute risk, they support careful clinical vigilance during gemcitabine–warfarin co-administration, including the period shortly after gemcitabine initiation, while recognising the limited availability of time-to-onset data. Prospective cohort studies incorporating INR monitoring, warfarin dose information, and defined exposure denominators are needed to validate this signal and to estimate the absolute clinical risk. A focused mechanistic research agenda would help to clarify the basis of this warfarin-specific signal. Priorities include prospective pharmacokinetic–pharmacodynamic studies in gemcitabine-treated patients with serial INR, warfarin dose, albumin, hepatic function, and inflammatory markers, ideally with measurement of S- and R-warfarin concentrations and *CYP2C9/VKORC1* genotyping; *in vitro* assessment of the effect of gemcitabine and its principal metabolite (2′,2′-difluorodeoxyuridine) on CYP2C9-mediated warfarin metabolism and on protein binding; population-pharmacokinetic modelling of warfarin across gemcitabine treatment cycles; and comparative real-world cohort studies that use the direct oral anticoagulants as an active comparator, with exposure denominators, to test compound specificity and to quantify absolute risk.

## Data Availability

The data analyzed in this study is subject to the following licenses/restrictions: The data used in this study were obtained from VigiBase, the WHO global database of individual case safety reports, maintained by the Uppsala Monitoring Centre (UMC). These data are not publicly available and were accessed under permission for the present study. Because the authors do not own or control the underlying VigiBase data, they cannot share the dataset. Access may be requested from the UMC and is subject to its review and approval under the WHO Programme for International Drug Monitoring. Requests to access these datasets should be directed to Uppsala Monitoring Centre (UMC) VigiBase data access: https://who-umc.org/vigibase-data-access/.
