# Supplementary material for: Haemorrhagic event reporting with concomitant gemcitabine and warfarin: a VigiBase disproportionality analysis
Source: Front Pharmacol. 2026 Sep 10;17:1858594. doi: 10.3389/fphar.2026.1858594 (PMC13601313; doi:10.3389/fphar.2026.1858594)
Supplement: Supplementary file 1 [file Table1.docx]

| **Supplementary Table 1.  Preferred terms included in the Haemorrhages Standardised MedDRA Queries** | |
| --- | --- |
| **Term** | **Classification** |
| Blood urine | PT narrow |
| Blood urine present | PT narrow |
| Gastric occult blood positive | PT narrow |
| Plasmin increased | PT narrow |
| Prekallikrein increased | PT narrow |
| Thrombomodulin increased | PT narrow |
| Urinary occult blood | PT narrow |
| Urinary occult blood positive | PT narrow |
| Abnormal clotting factor | PT broad |
| Acquired dysfibrinogenaemia | PT broad |
| Acquired factor IX deficiency | PT broad |
| Acquired factor V deficiency | PT broad |
| Acquired factor VIII deficiency | PT broad |
| Acquired factor XI deficiency | PT broad |
| Acquired haemophilia | PT broad |
| Acquired Von Willebrand's disease | PT broad |
| Activated partial thromboplastin time abnormal | PT broad |
| Activated partial thromboplastin time prolonged | PT broad |
| Activated partial thromboplastin time ratio abnormal | PT broad |
| Activated partial thromboplastin time ratio fluctuation | PT broad |
| Activated partial thromboplastin time ratio increased | PT broad |
| Anti factor IX antibody positive | PT broad |
| Anti factor V antibody positive | PT broad |
| Anti factor VII antibody positive | PT broad |
| Anti factor VIII antibody positive | PT broad |
| Anti factor X activity abnormal | PT broad |
| Anti factor X activity increased | PT broad |
| Anti factor X antibody positive | PT broad |
| Anti factor XI antibody positive | PT broad |
| Anti factor XII antibody positive | PT broad |
| Antithrombin III abnormal | PT broad |
| Antithrombin III increased | PT broad |
| Bleeding time abnormal | PT broad |
| Bleeding time prolonged | PT broad |
| Blood fibrinogen abnormal | PT broad |
| Blood fibrinogen decreased | PT broad |
| Blood thrombin abnormal | PT broad |
| Blood thrombin decreased | PT broad |
| Blood thromboplastin abnormal | PT broad |
| Blood thromboplastin decreased | PT broad |
| Capillary fragility abnormal | PT broad |
| Capillary fragility increased | PT broad |
| Capillary permeability increased | PT broad |
| Circulating anticoagulant | PT broad |
| Clot retraction abnormal | PT broad |
| Clot retraction time prolonged | PT broad |
| Coagulation disorder neonatal | PT broad |
| Coagulation factor decreased | PT broad |
| Coagulation factor deficiency | PT broad |
| Coagulation factor IX level abnormal | PT broad |
| Coagulation factor IX level decreased | PT broad |
| Coagulation factor level abnormal | PT broad |
| Coagulation factor V level abnormal | PT broad |
| Coagulation factor V level decreased | PT broad |
| Coagulation factor VII level abnormal | PT broad |
| Coagulation factor VII level decreased | PT broad |
| Coagulation factor VIII level abnormal | PT broad |
| Coagulation factor VIII level decreased | PT broad |
| Coagulation factor X level abnormal | PT broad |
| Coagulation factor X level decreased | PT broad |
| Coagulation factor XI level abnormal | PT broad |
| Coagulation factor XI level decreased | PT broad |
| Coagulation factor XII level abnormal | PT broad |
| Coagulation factor XII level decreased | PT broad |
| Coagulation factor XIII level abnormal | PT broad |
| Coagulation factor XIII level decreased | PT broad |
| Coagulation time abnormal | PT broad |
| Coagulation time prolonged | PT broad |
| Coagulopathy | PT broad |
| Dilutional coagulopathy | PT broad |
| Ethanol gelation test positive | PT broad |
| Factor IX inhibition | PT broad |
| Factor VII activity decreased | PT broad |
| Factor VIII activity abnormal | PT broad |
| Factor VIII activity decreased | PT broad |
| Factor VIII inhibition | PT broad |
| Factor XII activity decreased | PT broad |
| Fibrin abnormal | PT broad |
| Fibrin D dimer decreased | PT broad |
| Fibrin D dimer increased | PT broad |
| Fibrin decreased | PT broad |
| Fibrin degradation products | PT broad |
| Fibrin degradation products increased | PT broad |
| Fibrinogen degradation products increased | PT broad |
| Fibrinolysis abnormal | PT broad |
| Fibrinolysis increased | PT broad |
| Haematocrit abnormal | PT broad |
| Haematocrit decreased | PT broad |
| Haemoglobin abnormal | PT broad |
| Haemoglobin decreased | PT broad |
| Haemosiderin urine positive | PT broad |
| Hypocoagulable state | PT broad |
| Hypofibrinogenaemia | PT broad |
| Hypoprothrombinaemia | PT broad |
| Hypothrombinaemia | PT broad |
| Hypothromboplastinaemia | PT broad |
| International normalised ratio abnormal | PT broad |
| International normalised ratio increased | PT broad |
| ISTH score for disseminated intravascular coagulation | PT broad |
| Plasmin decreased | PT broad |
| Plasmin inhibitor increased | PT broad |
| Plasminogen activator inhibitor | PT broad |
| Plasminogen activator inhibitor decreased | PT broad |
| Plasminogen decreased | PT broad |
| Plasminogen increased | PT broad |
| Platelet factor 4 decreased | PT broad |
| Protein C increased | PT broad |
| Protein S abnormal | PT broad |
| Protein S increased | PT broad |
| Prothrombin fragment 1.2 increased | PT broad |
| Prothrombin level abnormal | PT broad |
| Prothrombin level decreased | PT broad |
| Prothrombin time abnormal | PT broad |
| Prothrombin time prolonged | PT broad |
| Prothrombin time ratio abnormal | PT broad |
| Prothrombin time ratio increased | PT broad |
| Red blood cell count decreased | PT broad |
| Reticulocyte count increased | PT broad |
| Reticulocyte percentage abnormal | PT broad |
| Reticulocyte percentage increased | PT broad |
| Russell's viper venom time abnormal | PT broad |
| Septic coagulopathy | PT broad |
| Thrombin time abnormal | PT broad |
| Thrombin time prolonged | PT broad |
| Thrombin-antithrombin III complex abnormal | PT broad |
| Thrombin-antithrombin III complex increased | PT broad |
| Von Willebrand's factor antibody positive | PT broad |
| Von Willebrand's factor multimers abnormal | PT broad |
| Abdominal wall haematoma | PT narrow |
| Abdominal wall haemorrhage | PT narrow |
| Abnormal menstrual clots | PT narrow |
| Abnormal uterine bleeding | PT narrow |
| Abnormal withdrawal bleeding | PT narrow |
| Achenbach syndrome | PT narrow |
| Acute haemorrhagic leukoencephalitis | PT narrow |
| Acute haemorrhagic ulcerative colitis | PT narrow |
| Administration site bruise | PT narrow |
| Administration site haematoma | PT narrow |
| Administration site haemorrhage | PT narrow |
| Adrenal haematoma | PT narrow |
| Adrenal haemorrhage | PT narrow |
| Anal fissure haemorrhage | PT narrow |
| Anal haemorrhage | PT narrow |
| Anal ulcer haemorrhage | PT narrow |
| Anastomotic haemorrhage | PT narrow |
| Anastomotic ulcer haemorrhage | PT narrow |
| Aneurysm ruptured | PT narrow |
| Angina bullosa haemorrhagica | PT narrow |
| Anorectal varices haemorrhage | PT narrow |
| Anticoagulant-related nephropathy | PT narrow |
| Antiplatelet reversal therapy | PT narrow |
| Aortic aneurysm rupture | PT narrow |
| Aortic dissection rupture | PT narrow |
| Aortic intramural haematoma | PT narrow |
| Aortic perforation | PT narrow |
| Aortic rupture | PT narrow |
| Aponeurosis contusion | PT narrow |
| Application site bruise | PT narrow |
| Application site haematoma | PT narrow |
| Application site haemorrhage | PT narrow |
| Application site purpura | PT narrow |
| Arterial haemorrhage | PT narrow |
| Arterial intramural haematoma | PT narrow |
| Arterial perforation | PT narrow |
| Arterial rupture | PT narrow |
| Arteriovenous fistula site haematoma | PT narrow |
| Arteriovenous fistula site haemorrhage | PT narrow |
| Arteriovenous graft site haematoma | PT narrow |
| Arteriovenous graft site haemorrhage | PT narrow |
| Astringent therapy | PT narrow |
| Atrial rupture | PT narrow |
| Auricular haematoma | PT narrow |
| Basal ganglia haematoma | PT narrow |
| Basal ganglia haemorrhage | PT narrow |
| Basilar artery perforation | PT narrow |
| Bladder tamponade | PT narrow |
| Bleeding varicose vein | PT narrow |
| Blood blister | PT narrow |
| Blood loss anaemia | PT narrow |
| Blood urine | PT narrow |
| Blood urine present | PT narrow |
| Bloody discharge | PT narrow |
| Bloody peritoneal effluent | PT narrow |
| Bone contusion | PT narrow |
| Bone marrow haemorrhage | PT narrow |
| Brain contusion | PT narrow |
| Brain stem haematoma | PT narrow |
| Brain stem haemorrhage | PT narrow |
| Brain stem microhaemorrhage | PT narrow |
| Breast haematoma | PT narrow |
| Breast haemorrhage | PT narrow |
| Broad ligament haematoma | PT narrow |
| Bronchial haemorrhage | PT narrow |
| Bronchial varices haemorrhage | PT narrow |
| Bullous haemorrhagic dermatosis | PT narrow |
| Bursal haematoma | PT narrow |
| Cardiac contusion | PT narrow |
| Carotid aneurysm rupture | PT narrow |
| Carotid artery perforation | PT narrow |
| Carotid blowout syndrome | PT narrow |
| Catheter site bruise | PT narrow |
| Catheter site haematoma | PT narrow |
| Catheter site haemorrhage | PT narrow |
| Central nervous system haemorrhage | PT narrow |
| Cephalhaematoma | PT narrow |
| Cerebellar haematoma | PT narrow |
| Cerebellar haemorrhage | PT narrow |
| Cerebellar microhaemorrhage | PT narrow |
| Cerebral aneurysm perforation | PT narrow |
| Cerebral aneurysm ruptured syphilitic | PT narrow |
| Cerebral arteriovenous malformation haemorrhagic | PT narrow |
| Cerebral artery perforation | PT narrow |
| Cerebral cyst haemorrhage | PT narrow |
| Cerebral haematoma | PT narrow |
| Cerebral haemorrhage | PT narrow |
| Cerebral haemorrhage foetal | PT narrow |
| Cerebral haemorrhage neonatal | PT narrow |
| Cerebral microhaemorrhage | PT narrow |
| Cervix haematoma uterine | PT narrow |
| Cervix haemorrhage uterine | PT narrow |
| Chest wall haematoma | PT narrow |
| Choroidal haematoma | PT narrow |
| Choroidal haemorrhage | PT narrow |
| Chronic gastrointestinal bleeding | PT narrow |
| Chronic pigmented purpura | PT narrow |
| Ciliary body haemorrhage | PT narrow |
| Coital bleeding | PT narrow |
| Colonic haematoma | PT narrow |
| Conjunctival haemorrhage | PT narrow |
| Contusion | PT narrow |
| Corneal bleeding | PT narrow |
| Cullen's sign | PT narrow |
| Cystitis haemorrhagic | PT narrow |
| Deep dissecting haematoma | PT narrow |
| Diarrhoea haemorrhagic | PT narrow |
| Disseminated intravascular coagulation | PT narrow |
| Diverticulitis intestinal haemorrhagic | PT narrow |
| Diverticulum intestinal haemorrhagic | PT narrow |
| Duodenal ulcer haemorrhage | PT narrow |
| Duodenitis haemorrhagic | PT narrow |
| Ear haemorrhage | PT narrow |
| Ecchymosis | PT narrow |
| Encephalitis haemorrhagic | PT narrow |
| Enterocolitis haemorrhagic | PT narrow |
| Epidural haemorrhage | PT narrow |
| Epistaxis | PT narrow |
| Exsanguination | PT narrow |
| Extra-axial haemorrhage | PT narrow |
| Extradural haematoma | PT narrow |
| Extradural haematoma evacuation | PT narrow |
| Extravasation blood | PT narrow |
| Eye contusion | PT narrow |
| Eye haematoma | PT narrow |
| Eye haemorrhage | PT narrow |
| Eyelid bleeding | PT narrow |
| Eyelid contusion | PT narrow |
| Eyelid haematoma | PT narrow |
| Femoral artery perforation | PT narrow |
| Femoral vein perforation | PT narrow |
| Foetal-maternal haemorrhage | PT narrow |
| Fothergill sign positive | PT narrow |
| Gallbladder haematoma | PT narrow |
| Gastric haemorrhage | PT narrow |
| Gastric ulcer haemorrhage | PT narrow |
| Gastric ulcer haemorrhage, obstructive | PT narrow |
| Gastric varices haemorrhage | PT narrow |
| Gastritis alcoholic haemorrhagic | PT narrow |
| Gastritis haemorrhagic | PT narrow |
| Gastroduodenal haemorrhage | PT narrow |
| Gastrointestinal anastomotic haemorrhage | PT narrow |
| Gastrointestinal haemorrhage | PT narrow |
| Gastrointestinal polyp haemorrhage | PT narrow |
| Gastrointestinal ulcer haemorrhage | PT narrow |
| Gastrointestinal vascular malformation haemorrhagic | PT narrow |
| Gastrooesophageal haemorrhage | PT narrow |
| Genital contusion | PT narrow |
| Genital haemorrhage | PT narrow |
| Gingival bleeding | PT narrow |
| Graft haemorrhage | PT narrow |
| Grey Turner's sign | PT narrow |
| Haemangioma rupture | PT narrow |
| Haemarthrosis | PT narrow |
| Haematemesis | PT narrow |
| Haematochezia | PT narrow |
| Haematocoele | PT narrow |
| Haematoma | PT narrow |
| Haematoma evacuation | PT narrow |
| Haematoma infection | PT narrow |
| Haematoma muscle | PT narrow |
| Haematosalpinx | PT narrow |
| Haematospermia | PT narrow |
| Haematotympanum | PT narrow |
| Haematuria | PT narrow |
| Haematuria traumatic | PT narrow |
| Haemobilia | PT narrow |
| Haemoperitoneum | PT narrow |
| Haemophilic arthropathy | PT narrow |
| Haemophilic pseudotumour | PT narrow |
| Haemoptysis | PT narrow |
| Haemorrhage | PT narrow |
| Haemorrhage coronary artery | PT narrow |
| Haemorrhage foetal | PT narrow |
| Haemorrhage in pregnancy | PT narrow |
| Haemorrhage intracranial | PT narrow |
| Haemorrhage neonatal | PT narrow |
| Haemorrhage subcutaneous | PT narrow |
| Haemorrhage subepidermal | PT narrow |
| Haemorrhage urinary tract | PT narrow |
| Haemorrhagic adrenal infarction | PT narrow |
| Haemorrhagic arteriovenous malformation | PT narrow |
| Haemorrhagic ascites | PT narrow |
| Haemorrhagic breast cyst | PT narrow |
| Haemorrhagic cerebellar infarction | PT narrow |
| Haemorrhagic cerebral infarction | PT narrow |
| Haemorrhagic cyst | PT narrow |
| Haemorrhagic diathesis | PT narrow |
| Haemorrhagic disease of newborn | PT narrow |
| Haemorrhagic disorder | PT narrow |
| Haemorrhagic erosive gastritis | PT narrow |
| Haemorrhagic gastroenteritis | PT narrow |
| Haemorrhagic hepatic cyst | PT narrow |
| Haemorrhagic infarction | PT narrow |
| Haemorrhagic intestinal infarction | PT narrow |
| Haemorrhagic necrotic pancreatitis | PT narrow |
| Haemorrhagic occlusive retinal vasculitis | PT narrow |
| Haemorrhagic ovarian cyst | PT narrow |
| Haemorrhagic pancreatic cyst | PT narrow |
| Haemorrhagic stroke | PT narrow |
| Haemorrhagic thyroid cyst | PT narrow |
| Haemorrhagic transformation stroke | PT narrow |
| Haemorrhagic tumour necrosis | PT narrow |
| Haemorrhagic urticaria | PT narrow |
| Haemorrhagic vasculitis | PT narrow |
| Haemorrhoidal haemorrhage | PT narrow |
| Haemostasis | PT narrow |
| Haemothorax | PT narrow |
| Heavy menstrual bleeding | PT narrow |
| Henoch-Schonlein purpura | PT narrow |
| Hepatic artery haemorrhage | PT narrow |
| Hepatic haemangioma rupture | PT narrow |
| Hepatic haematoma | PT narrow |
| Hepatic haemorrhage | PT narrow |
| Hereditary haemorrhagic telangiectasia | PT narrow |
| Hyperfibrinolysis | PT narrow |
| Hypergammaglobulinaemic purpura of Waldenstrom | PT narrow |
| Hyphaema | PT narrow |
| Iliac artery perforation | PT narrow |
| Iliac artery rupture | PT narrow |
| Iliac vein perforation | PT narrow |
| Immune thrombocytopenia | PT narrow |
| Implant site bruising | PT narrow |
| Implant site haematoma | PT narrow |
| Implant site haemorrhage | PT narrow |
| Incision site haematoma | PT narrow |
| Incision site haemorrhage | PT narrow |
| Increased tendency to bruise | PT narrow |
| Induced abortion haemorrhage | PT narrow |
| Inferior vena cava perforation | PT narrow |
| Infusion site bruising | PT narrow |
| Infusion site haematoma | PT narrow |
| Infusion site haemorrhage | PT narrow |
| Injection site bruising | PT narrow |
| Injection site haematoma | PT narrow |
| Injection site haemorrhage | PT narrow |
| Instillation site bruise | PT narrow |
| Instillation site haematoma | PT narrow |
| Instillation site haemorrhage | PT narrow |
| Intermenstrual bleeding | PT narrow |
| Internal haemorrhage | PT narrow |
| Intestinal haematoma | PT narrow |
| Intestinal haemorrhage | PT narrow |
| Intestinal varices haemorrhage | PT narrow |
| Intra-abdominal haematoma | PT narrow |
| Intra-abdominal haemorrhage | PT narrow |
| Intracerebral haematoma evacuation | PT narrow |
| Intracranial haematoma | PT narrow |
| Intracranial haemorrhage neonatal | PT narrow |
| Intracranial tumour haemorrhage | PT narrow |
| Intramyocardial dissecting haematoma | PT narrow |
| Intraocular haematoma | PT narrow |
| Intrapartum haemorrhage | PT narrow |
| Intratumoural haematoma | PT narrow |
| Intraventricular haemorrhage | PT narrow |
| Intraventricular haemorrhage neonatal | PT narrow |
| Iris haemorrhage | PT narrow |
| Joint microhaemorrhage | PT narrow |
| Jugular vein haemorrhage | PT narrow |
| Kidney contusion | PT narrow |
| Lacrimal haemorrhage | PT narrow |
| Large intestinal haemorrhage | PT narrow |
| Large intestinal ulcer haemorrhage | PT narrow |
| Laryngeal haematoma | PT narrow |
| Laryngeal haemorrhage | PT narrow |
| Lip haematoma | PT narrow |
| Lip haemorrhage | PT narrow |
| Liver contusion | PT narrow |
| Lower gastrointestinal haemorrhage | PT narrow |
| Lower limb artery perforation | PT narrow |
| Lymph node haemorrhage | PT narrow |
| Mallory-Weiss syndrome | PT narrow |
| Mediastinal haematoma | PT narrow |
| Mediastinal haemorrhage | PT narrow |
| Medical device site bruise | PT narrow |
| Medical device site haematoma | PT narrow |
| Medical device site haemorrhage | PT narrow |
| Melaena | PT narrow |
| Melaena neonatal | PT narrow |
| Meningorrhagia | PT narrow |
| Menometrorrhagia | PT narrow |
| Menstrual clots | PT narrow |
| Mesenteric haematoma | PT narrow |
| Mesenteric haemorrhage | PT narrow |
| Mouth haemorrhage | PT narrow |
| Mucocutaneous haemorrhage | PT narrow |
| Mucosal haemorrhage | PT narrow |
| Muscle contusion | PT narrow |
| Muscle haemorrhage | PT narrow |
| Myocardial haemorrhage | PT narrow |
| Myocardial rupture | PT narrow |
| Naevus haemorrhage | PT narrow |
| Nail bed bleeding | PT narrow |
| Nasal septum haematoma | PT narrow |
| Neonatal gastrointestinal haemorrhage | PT narrow |
| Nephritis haemorrhagic | PT narrow |
| Nipple exudate bloody | PT narrow |
| Ocular retrobulbar haemorrhage | PT narrow |
| Oesophageal haemorrhage | PT narrow |
| Oesophageal intramural haematoma | PT narrow |
| Oesophageal ulcer haemorrhage | PT narrow |
| Oesophageal varices haemorrhage | PT narrow |
| Oesophagitis haemorrhagic | PT narrow |
| Omental haemorrhage | PT narrow |
| Optic disc haemorrhage | PT narrow |
| Optic nerve sheath haemorrhage | PT narrow |
| Oral blood blister | PT narrow |
| Oral contusion | PT narrow |
| Oral mucosa haematoma | PT narrow |
| Oral purpura | PT narrow |
| Orbital haematoma | PT narrow |
| Orbital haemorrhage | PT narrow |
| Osteorrhagia | PT narrow |
| Ovarian haematoma | PT narrow |
| Ovarian haemorrhage | PT narrow |
| Palpable purpura | PT narrow |
| Pancreatic haemorrhage | PT narrow |
| Pancreatic pseudocyst haemorrhage | PT narrow |
| Pancreatitis haemorrhagic | PT narrow |
| Papillary muscle haemorrhage | PT narrow |
| Paranasal sinus haematoma | PT narrow |
| Paranasal sinus haemorrhage | PT narrow |
| Parathyroid haemorrhage | PT narrow |
| Parotid gland haemorrhage | PT narrow |
| Pelvic haematoma | PT narrow |
| Pelvic haematoma obstetric | PT narrow |
| Pelvic haemorrhage | PT narrow |
| Penile contusion | PT narrow |
| Penile haematoma | PT narrow |
| Penile haemorrhage | PT narrow |
| Peptic ulcer haemorrhage | PT narrow |
| Pericardial haemorrhage | PT narrow |
| Perineal haematoma | PT narrow |
| Periorbital haematoma | PT narrow |
| Periorbital haemorrhage | PT narrow |
| Periosteal haematoma | PT narrow |
| Peripartum haemorrhage | PT narrow |
| Peripheral artery aneurysm rupture | PT narrow |
| Peripheral exudative haemorrhagic chorioretinopathy | PT narrow |
| Peripheral vascular haematoma | PT narrow |
| Peritoneal haematoma | PT narrow |
| Periventricular haemorrhage neonatal | PT narrow |
| Petechiae | PT narrow |
| Pharyngeal contusion | PT narrow |
| Pharyngeal haematoma | PT narrow |
| Pharyngeal haemorrhage | PT narrow |
| Pituitary apoplexy | PT narrow |
| Pituitary haemorrhage | PT narrow |
| Placenta praevia haemorrhage | PT narrow |
| Polymenorrhagia | PT narrow |
| Post abortion haemorrhage | PT narrow |
| Post procedural contusion | PT narrow |
| Post procedural haematoma | PT narrow |
| Post procedural haematuria | PT narrow |
| Post procedural haemorrhage | PT narrow |
| Post transfusion purpura | PT narrow |
| Postmenopausal haemorrhage | PT narrow |
| Postpartum haemorrhage | PT narrow |
| Post-traumatic punctate intraepidermal haemorrhage | PT narrow |
| Premature separation of placenta | PT narrow |
| Procedural haemorrhage | PT narrow |
| Proctitis haemorrhagic | PT narrow |
| Prostatic haemorrhage | PT narrow |
| Pulmonary alveolar haemorrhage | PT narrow |
| Pulmonary contusion | PT narrow |
| Pulmonary haematoma | PT narrow |
| Pulmonary haemorrhage | PT narrow |
| Pulmonary haemorrhage neonatal | PT narrow |
| Puncture site bruise | PT narrow |
| Puncture site haematoma | PT narrow |
| Puncture site haemorrhage | PT narrow |
| Purpura | PT narrow |
| Purpura fulminans | PT narrow |
| Purpura neonatal | PT narrow |
| Purpura non-thrombocytopenic | PT narrow |
| Purpura senile | PT narrow |
| Putamen haemorrhage | PT narrow |
| Radiation associated haemorrhage | PT narrow |
| Rectal haemorrhage | PT narrow |
| Rectal ulcer haemorrhage | PT narrow |
| Renal artery perforation | PT narrow |
| Renal cyst haemorrhage | PT narrow |
| Renal haematoma | PT narrow |
| Renal haemorrhage | PT narrow |
| Respiratory tract haemorrhage | PT narrow |
| Respiratory tract haemorrhage neonatal | PT narrow |
| Retinal aneurysm rupture | PT narrow |
| Retinal haemorrhage | PT narrow |
| Retinopathy haemorrhagic | PT narrow |
| Retroperitoneal haematoma | PT narrow |
| Retroperitoneal haemorrhage | PT narrow |
| Retroplacental haematoma | PT narrow |
| Ruptured cerebral aneurysm | PT narrow |
| Scalp haematoma | PT narrow |
| Scleral haematoma | PT narrow |
| Scleral haemorrhage | PT narrow |
| Scrotal haematocoele | PT narrow |
| Scrotal haematoma | PT narrow |
| Scrotal haemorrhage | PT narrow |
| Shock haemorrhagic | PT narrow |
| Skin haemorrhage | PT narrow |
| Skin neoplasm bleeding | PT narrow |
| Skin ulcer haemorrhage | PT narrow |
| Small intestinal haemorrhage | PT narrow |
| Small intestinal ulcer haemorrhage | PT narrow |
| Soft tissue haemorrhage | PT narrow |
| Spermatic cord haemorrhage | PT narrow |
| Spinal cord haematoma | PT narrow |
| Spinal cord haemorrhage | PT narrow |
| Spinal epidural haematoma | PT narrow |
| Spinal epidural haemorrhage | PT narrow |
| Spinal subarachnoid haemorrhage | PT narrow |
| Spinal subdural haematoma | PT narrow |
| Spinal subdural haemorrhage | PT narrow |
| Spleen contusion | PT narrow |
| Splenic artery perforation | PT narrow |
| Splenic haematoma | PT narrow |
| Splenic haemorrhage | PT narrow |
| Splenic varices haemorrhage | PT narrow |
| Splinter haemorrhages | PT narrow |
| Spontaneous haematoma | PT narrow |
| Spontaneous haemorrhage | PT narrow |
| Spontaneous subdural haematoma | PT narrow |
| Stoma site haemorrhage | PT narrow |
| Stomatitis haemorrhagic | PT narrow |
| Subarachnoid haematoma | PT narrow |
| Subarachnoid haemorrhage | PT narrow |
| Subarachnoid haemorrhage neonatal | PT narrow |
| Subcapsular hepatic haematoma | PT narrow |
| Subcapsular renal haematoma | PT narrow |
| Subcapsular splenic haematoma | PT narrow |
| Subchorionic haematoma | PT narrow |
| Subchorionic haemorrhage | PT narrow |
| Subclavian artery perforation | PT narrow |
| Subclavian vein perforation | PT narrow |
| Subcutaneous haematoma | PT narrow |
| Subdural haematoma | PT narrow |
| Subdural haematoma evacuation | PT narrow |
| Subdural haemorrhage | PT narrow |
| Subdural haemorrhage neonatal | PT narrow |
| Subendocardial haemorrhage | PT narrow |
| Subfascial haematoma | PT narrow |
| Subgaleal haematoma | PT narrow |
| Subgaleal haemorrhage | PT narrow |
| Subretinal haematoma | PT narrow |
| Superior vena cava perforation | PT narrow |
| Terson syndrome | PT narrow |
| Testicular haemorrhage | PT narrow |
| Thalamic microhaemorrhage | PT narrow |
| Thalamus haemorrhage | PT narrow |
| Third stage postpartum haemorrhage | PT narrow |
| Thoracic haemorrhage | PT narrow |
| Thrombocytopenic purpura | PT narrow |
| Thrombotic thrombocytopenic purpura | PT narrow |
| Thyroid haemorrhage | PT narrow |
| Tongue haematoma | PT narrow |
| Tongue haemorrhage | PT narrow |
| Tonsillar haemorrhage | PT narrow |
| Tooth pulp haemorrhage | PT narrow |
| Tooth socket haemorrhage | PT narrow |
| Tracheal haemorrhage | PT narrow |
| Traumatic haematoma | PT narrow |
| Traumatic haemorrhage | PT narrow |
| Traumatic haemothorax | PT narrow |
| Traumatic intracranial haematoma | PT narrow |
| Traumatic intracranial haemorrhage | PT narrow |
| Traumatic subarachnoid haemorrhage | PT narrow |
| Tumour haemorrhage | PT narrow |
| Ulcer haemorrhage | PT narrow |
| Umbilical cord haemorrhage | PT narrow |
| Umbilical haematoma | PT narrow |
| Umbilical haemorrhage | PT narrow |
| Unexpected vaginal bleeding on hormonal IUD | PT narrow |
| Upper gastrointestinal haemorrhage | PT narrow |
| Ureteric haemorrhage | PT narrow |
| Urethral haemorrhage | PT narrow |
| Urinary bladder clot evacuation | PT narrow |
| Urinary bladder haematoma | PT narrow |
| Urinary bladder haemorrhage | PT narrow |
| Urogenital haemorrhage | PT narrow |
| Uterine haematoma | PT narrow |
| Uterine haemorrhage | PT narrow |
| Vaccination site bruising | PT narrow |
| Vaccination site haematoma | PT narrow |
| Vaccination site haemorrhage | PT narrow |
| Vaginal haematoma | PT narrow |
| Vaginal haemorrhage | PT narrow |
| Varicose vein ruptured | PT narrow |
| Vascular access site bruising | PT narrow |
| Vascular access site haematoma | PT narrow |
| Vascular access site haemorrhage | PT narrow |
| Vascular access site rupture | PT narrow |
| Vascular anastomotic haemorrhage | PT narrow |
| Vascular graft haemorrhage | PT narrow |
| Vascular pseudoaneurysm ruptured | PT narrow |
| Vascular purpura | PT narrow |
| Vascular rupture | PT narrow |
| Vein rupture | PT narrow |
| Venous haemorrhage | PT narrow |
| Venous perforation | PT narrow |
| Ventricle rupture | PT narrow |
| Vertebral artery perforation | PT narrow |
| Vessel puncture site bruise | PT narrow |
| Vessel puncture site haematoma | PT narrow |
| Vessel puncture site haemorrhage | PT narrow |
| Vitreous haematoma | PT narrow |
| Vitreous haemorrhage | PT narrow |
| Vocal cord haemorrhage | PT narrow |
| Vulval haematoma | PT narrow |
| Vulval haematoma evacuation | PT narrow |
| Vulval haemorrhage | PT narrow |
| Withdrawal bleed | PT narrow |
| Wound haematoma | PT narrow |
| Wound haemorrhage | PT narrow |

| **Supplementary Table 2: Top 20 Reported Indications for Gemcitabine by Anticoagulant Sub-group** | | | | | | | | | |
| --- | --- | --- | --- | --- | --- | --- | --- | --- | --- |
| **Indication (MedDRA HLT)** | **All Cases** | **Warfarin** | **Acenocoumarol** | **Phenprocoumon** | **Fluindione** | **Rivaroxaban** | **Apixaban** | **Edoxaban** | **Dabigatran** |
| Unknown | 53,364 | 295 | 12 | 6 | 22 | 64 | 61 | 18 | 11 |
| Pancreatic neoplasms | 14,137 | 123 | 6 | 15 | 4 | 78 | 93 | 32 | 12 |
| Malignant hepatobiliary neoplasms | 5,782 | 25 | 0 | 5 | 0 | 17 | 19 | 6 | 3 |
| Breast and nipple neoplasms malignant | 4,422 | 30 | 0 | 4 | 0 | 10 | 2 | 0 | 1 |
| Non-small cell neoplasms malignant of the respiratory tract cell type specified | 3,844 | 55 | 5 | 8 | 2 | 14 | 15 | 6 | 0 |
| Respiratory tract and pleural neoplasms malignant cell type unspecified NEC | 3,112 | 13 | 3 | 3 | 0 | 6 | 3 | 4 | 2 |
| Ovarian neoplasms malignant (excl germ cell) | 3,069 | 16 | 1 | 3 | 0 | 7 | 3 | 3 | 0 |
| Bladder neoplasms malignant | 3,023 | 29 | 3 | 1 | 3 | 6 | 17 | 5 | 0 |
| Therapeutic procedures NEC | 1,892 | 35 | 1 | 1 | 1 | 18 | 35 | 1 | 3 |
| Neoplasms malignant site unspecified NEC | 1,733 | 12 | 1 | 2 | 0 | 6 | 11 | 1 | 2 |
| Malignant musculoskeletal and connective tissue neoplasms | 1,184 | 9 | 0 | 0 | 0 | 7 | 6 | 0 | 0 |
| Urinary tract neoplasms malignant NEC | 1,162 | 10 | 1 | 0 | 0 | 5 | 7 | 3 | 0 |
| Diffuse large B-cell lymphomas | 1,135 | 3 | 0 | 0 | 0 | 9 | 14 | 5 | 1 |
| Chemotherapies | 1,110 | 7 | 1 | 1 | 0 | 6 | 4 | 4 | 3 |
| Hodgkin's disease NEC | 620 | 1 | 0 | 0 | 0 | 0 | 1 | 0 | 0 |
| Neoplasms unspecified malignancy and site unspecified NEC | 408 | 1 | 0 | 0 | 0 | 1 | 2 | 1 | 0 |
| Renal pelvis and ureter neoplasms malignant | 405 | 3 | 0 | 1 | 2 | 0 | 0 | 0 | 0 |
| Malignant intestinal neoplasms | 399 | 6 | 0 | 0 | 0 | 0 | 0 | 0 | 0 |
| Non-Hodgkin's lymphomas NEC | 363 | 1 | 2 | 0 | 0 | 0 | 0 | 0 | 1 |
| Renal neoplasms malignant | 355 | 9 | 0 | 1 | 0 | 2 | 0 | 0 | 1 |
| **Abbreviations:** **MedDRA** = Medical Dictionary for Regulatory Activities; **HLT** = High-Level Term; **NEC** = Not Elsewhere Classified; **N** = Number of cases. | | | | | | | | | |
| **Data Source:** Data were extracted from VigiBase, the World Health Organization (WHO) global database of individual case safety reports. Reported indications for drug use were mapped to the MedDRA High-Level Term (HLT) hierarchy to group similar clinical conditions. | | | | | | | | | |
| **Missing Data ("Unknown"):** The "Unknown" category represents cases where the indication for gemcitabine was not provided by the original reporter. Missing or incomplete data is a well-known characteristic of spontaneous pharmacovigilance reporting systems. | | | | | | | | | |
| **Methodology:** If a single case report contained multiple specific indications that mapped to the exact same MedDRA HLT (e.g., "Pancreatic cancer" and "Pancreatic carcinoma"), the case was only counted once within that HLT to avoid artificial duplication of patient counts. | | | | | | | | | |

**Supplementary Table 3. Drug-role sensitivity analysis of the gemcitabine × anticoagulant interaction, with correction for multiple testing.**

For each anticoagulant, the interaction reporting odds ratio (IROR) with 95% confidence interval is shown for the main analysis (gemcitabine considered in any role: suspected, concomitant or interacting) and for the sensitivity analysis (gemcitabine restricted to a suspected or interacting role). Raw two-sided p-values are reported together with Bonferroni-corrected p-values and Benjamini–Hochberg false-discovery-rate (FDR) q-values computed across the eight anticoagulants within each analysis. Warfarin (pre-specified primary comparison) is shown in bold.

*Panel A — All haemorrhagic events (N = 6,191,806)*

| Anticoagulant | Main IROR (95% CI) | Main p | Main Bonf. | Main FDR q | Sensitivity IROR (95% CI) | Sens. p | Sens. Bonf. | Sens. FDR q |
| --- | --- | --- | --- | --- | --- | --- | --- | --- |
| **Warfarin** | **1.31 (1.07–1.60)** | **0.010** | **0.081** | **0.081** | **1.28 (1.00–1.63)** | **0.046** | **0.369** | **0.188** |
| Acenocoumarol | 0.41 (0.15–1.18) | 0.099 | 0.792 | 0.158 | 0.45 (0.16–1.28) | 0.132 | 1.000 | 0.211 |
| Phenprocoumon | 1.34 (0.68–2.65) | 0.395 | 1.000 | 0.420 | 1.37 (0.67–2.78) | 0.390 | 1.000 | 0.445 |
| Fluindione | 0.70 (0.34–1.46) | 0.347 | 1.000 | 0.420 | 0.57 (0.25–1.33) | 0.196 | 1.000 | 0.261 |
| Rivaroxaban | 0.86 (0.60–1.24) | 0.420 | 1.000 | 0.420 | 0.61 (0.36–1.03) | 0.062 | 0.496 | 0.188 |
| Apixaban | 1.40 (0.98–2.00) | 0.068 | 0.540 | 0.135 | 1.17 (0.72–1.89) | 0.523 | 1.000 | 0.523 |
| Edoxaban | 1.74 (1.05–2.88) | 0.031 | 0.249 | 0.125 | 0.45 (0.16–1.25) | 0.125 | 1.000 | 0.211 |
| Dabigatran | 0.39 (0.15–1.03) | 0.057 | 0.455 | 0.135 | 0.26 (0.06–1.12) | 0.070 | 0.564 | 0.188 |

*Panel B — Serious haemorrhagic events (N = 6,171,120)*

| Anticoagulant | Main IROR (95% CI) | Main p | Main Bonf. | Main FDR q | Sensitivity IROR (95% CI) | Sens. p | Sens. Bonf. | Sens. FDR q |
| --- | --- | --- | --- | --- | --- | --- | --- | --- |
| **Warfarin** | **1.36 (1.09–1.69)** | **0.006** | **0.046** | **0.046** | **1.33 (1.02–1.72)** | **0.033** | **0.261** | **0.119** |
| Acenocoumarol | 0.25 (0.06–1.04) | 0.057 | 0.456 | 0.151 | 0.27 (0.06–1.14) | 0.074 | 0.593 | 0.119 |
| Phenprocoumon | 0.90 (0.40–2.03) | 0.792 | 1.000 | 0.792 | 1.02 (0.45–2.32) | 0.958 | 1.000 | 0.958 |
| Fluindione | 0.64 (0.29–1.41) | 0.269 | 1.000 | 0.350 | 0.57 (0.23–1.41) | 0.225 | 1.000 | 0.300 |
| Rivaroxaban | 0.71 (0.49–1.04) | 0.075 | 0.603 | 0.151 | 0.54 (0.32–0.91) | 0.021 | 0.169 | 0.119 |
| Apixaban | 1.23 (0.85–1.78) | 0.266 | 1.000 | 0.350 | 1.11 (0.68–1.80) | 0.679 | 1.000 | 0.775 |
| Edoxaban | 1.32 (0.77–2.26) | 0.307 | 1.000 | 0.350 | 0.31 (0.09–1.00) | 0.050 | 0.397 | 0.119 |
| Dabigatran | 0.36 (0.14–0.95) | 0.039 | 0.309 | 0.151 | 0.25 (0.06–1.08) | 0.063 | 0.502 | 0.119 |

Models were adjusted for age group, sex, WHO region, and co-reported antiplatelet agents, NSAIDs, systemic corticosteroids, SSRIs/SNRIs, fluoropyrimidines, taxanes and platinum-based agents. The sensitivity analysis excluded the 13,638 reports (12.7% of gemcitabine reports) in which gemcitabine was coded only as a concomitant drug; 495 of the 707 gemcitabine–warfarin co-exposed reports (70%) were retained. Bonf. = Bonferroni-corrected p-value; FDR = Benjamini–Hochberg false-discovery-rate q-value.

**Supplementary Table 4. Sensitivity analysis adjusting for calendar time.**

To assess whether secular changes in reporting could confound the association, the year in which each report first entered VigiBase was categorised into four periods and modelled as a covariate. Panel A shows the temporal distribution of the study population; Panel B shows the gemcitabine × anticoagulant interaction reporting odds ratio (IROR) with 95% confidence interval from the primary model and from the model additionally adjusted for calendar period, for all haemorrhagic events and for serious haemorrhagic events. Warfarin (pre-specified primary comparison) is shown in bold.

*Panel A – Temporal distribution of the study population by report period*

| **Report period** | **Oncology reports** | **Gemcitabine reports** | **Warfarin reports** | **Gemcitabine + warfarin** | **Haemorrhage, n (%)** |
| --- | --- | --- | --- | --- | --- |
| 1994–2005 | 349,292 | 7,706 | 6,549 | 220 | 26,981 (7.7) |
| 2006–2011 | 750,812 | 10,281 | 11,316 | 206 | 56,137 (7.5) |
| 2012–2017 | 2,628,974 | 28,485 | 25,316 | 218 | 157,201 (6.0) |
| 2018–2025 | 5,666,666 | 61,058 | 19,393 | 63 | 244,950 (4.3) |

*Panel B – Gemcitabine × anticoagulant interaction, primary vs calendar-period-adjusted*

*All haemorrhagic events (N = 6,191,806)*

| **Anticoagulant** | **Primary IROR (95% CI)** | **Period-adjusted IROR (95% CI)** | **Adj. p** | **Bonf. p** | **FDR q** |
| --- | --- | --- | --- | --- | --- |
| **Warfarin** | **1.31 (1.07–1.60)** | **1.24 (1.01–1.52)** | **0.041** | **0.325** | **0.108** |
| Acenocoumarol | 0.41 (0.15–1.18) | 0.40 (0.14–1.12) | 0.082 | 0.656 | 0.131 |
| Phenprocoumon | 1.34 (0.68–2.65) | 1.23 (0.62–2.43) | 0.550 | 1.000 | 0.575 |
| Fluindione | 0.70 (0.34–1.46) | 0.70 (0.34–1.45) | 0.335 | 1.000 | 0.446 |
| Rivaroxaban | 0.86 (0.60–1.24) | 0.90 (0.62–1.30) | 0.575 | 1.000 | 0.575 |
| Apixaban | 1.40 (0.98–2.00) | 1.47 (1.03–2.11) | 0.034 | 0.273 | 0.108 |
| Edoxaban | 1.74 (1.05–2.88) | 1.83 (1.10–3.02) | 0.019 | 0.154 | 0.108 |
| Dabigatran | 0.39 (0.15–1.03) | 0.41 (0.16–1.08) | 0.072 | 0.572 | 0.131 |

*Serious haemorrhagic events (N = 6,171,120)*

| **Anticoagulant** | **Primary IROR (95% CI)** | **Period-adjusted IROR (95% CI)** | **Adj. p** | **Bonf. p** | **FDR q** |
| --- | --- | --- | --- | --- | --- |
| **Warfarin** | **1.36 (1.09–1.69)** | **1.42 (1.14–1.77)** | **0.0016** | **0.013** | **0.013** |
| Acenocoumarol | 0.25 (0.06–1.04) | 0.23 (0.05–0.98) | 0.047 | 0.374 | 0.125 |
| Phenprocoumon | 0.90 (0.40–2.03) | 0.88 (0.38–2.00) | 0.756 | 1.000 | 0.756 |
| Fluindione | 0.64 (0.29–1.41) | 0.59 (0.27–1.30) | 0.192 | 1.000 | 0.286 |
| Rivaroxaban | 0.71 (0.49–1.04) | 0.73 (0.50–1.06) | 0.098 | 0.785 | 0.196 |
| Apixaban | 1.23 (0.85–1.78) | 1.26 (0.87–1.83) | 0.214 | 1.000 | 0.286 |
| Edoxaban | 1.32 (0.77–2.26) | 1.34 (0.78–2.29) | 0.286 | 1.000 | 0.327 |
| Dabigatran | 0.36 (0.14–0.95) | 0.38 (0.14–0.98) | 0.046 | 0.364 | 0.125 |

Oncology reports, gemcitabine, warfarin, and co-exposure counts in Panel A are numbers of reports; the haemorrhage percentage is calculated among all oncology reports within each period. Panel B models were adjusted for age group, sex, WHO region, co-reported antiplatelet agents, NSAIDs, systemic corticosteroids, SSRIs/SNRIs, fluoropyrimidines, taxanes and platinum-based agents, and – in the adjusted column – report period (1994–2005, 2006–2011, 2012–2017, 2018–2025). Adj. p = two-sided p-value from the period-adjusted model; Bonf. p = Bonferroni-corrected p-value; FDR q = Benjamini–Hochberg false-discovery-rate q-value, each computed across the eight anticoagulants within each outcome.
